# Supplementary material for: A genome and gene catalog of the aquatic microbiomes of the Tibetan Plateau
Source: Nat Commun. 2024 Feb 16;15:1438. doi: 10.1038/s41467-024-45895-8 (PMC10873407; doi:10.1038/s41467-024-45895-8)
Supplement: Supplementary file 1 — Supplementary Information [file 41467_2024_45895_MOESM1_ESM.pdf]

# Supplementary Figures

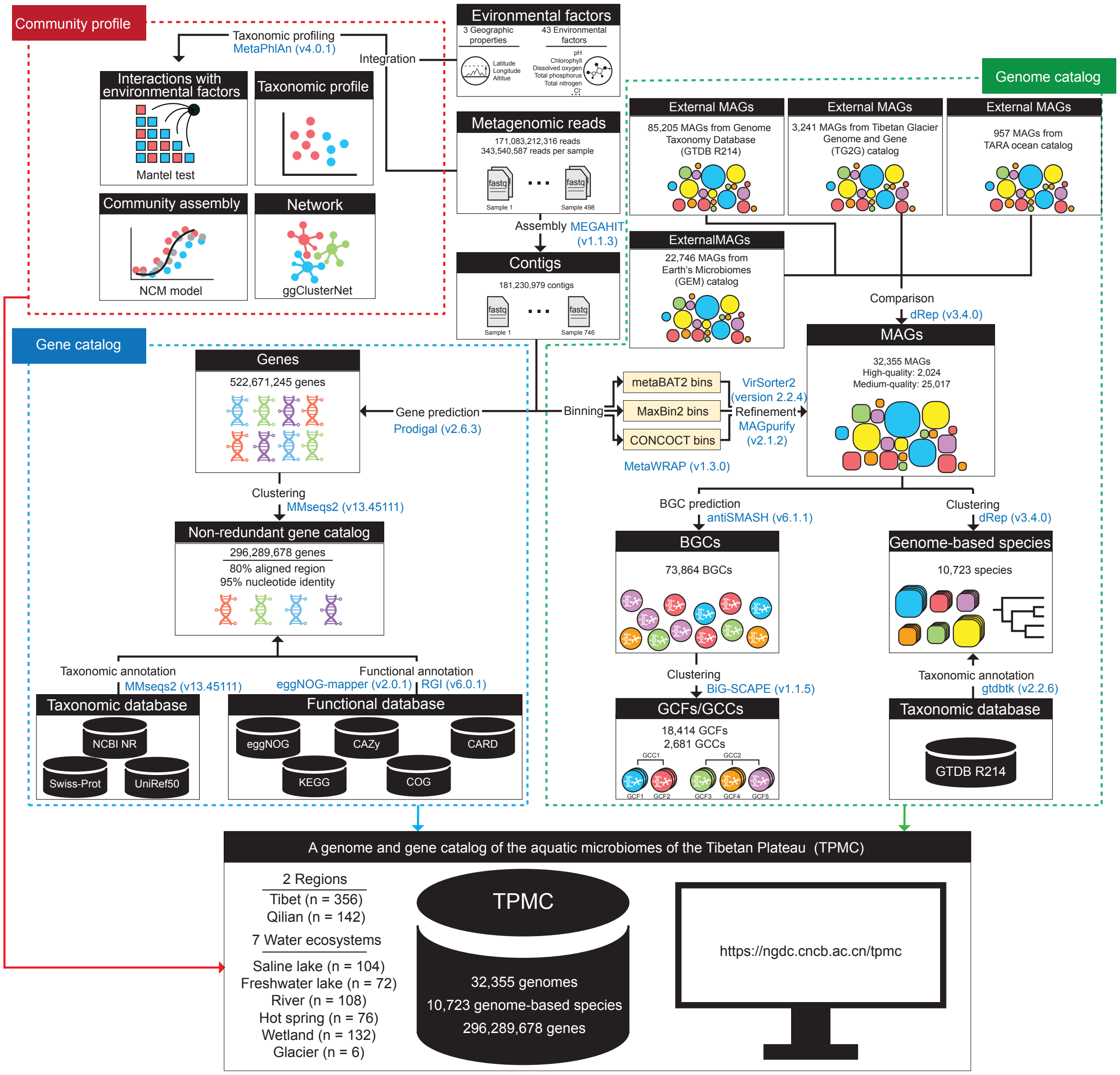


**Fig. S1 | Overview of the metagenomic analysis. a,** Quality-controlled, high-throughput DNA sequencing reads from Tibetan Plateau samples are used for 1) taxonomic profiling of the community and correlated with environmental factors, and then assembled into metagenomic contigs for: 2) establishment of the non-redundant gene catalog, annotated by taxonomic database and functional database; and 3) establishment of the metagenome-assembled genome catalog, with taxonomic annotations and prediction of biosynthetic gene clusters.

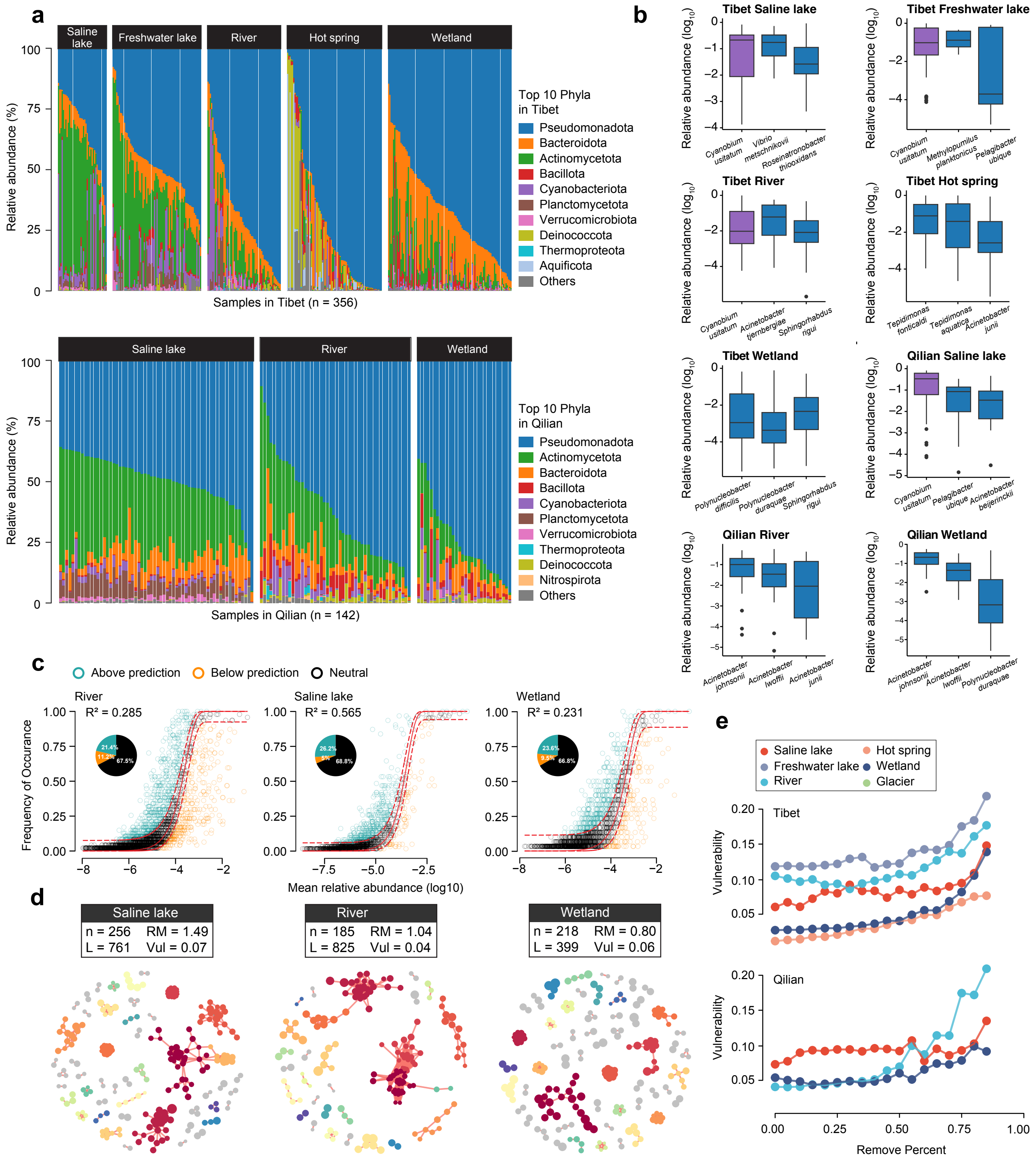


**Fig. S2 | Microbial composition, assemblage, and networks of the Tibetan Plateau microbial communities. a**, The relative abundances of the top 10 phyla across water ecosystems and regions. **b**, Boxplots show the relative abundances of the top 3 species across water ecosystems and regions. Boxes represent the interquartile range between the first and third quartiles and the line inside

represents the median. Whiskers denote the lowest and highest values within the 1.5×interquartile range from the first and third quartiles, respectively. **c**, Fit of the NCM of microbial community assemblage in three water ecosystems in Qilian. The solid red lines indicate the best fit to the NCM, and the dashed red lines represent 95% confidence intervals around the model prediction. Genera that occur more or less frequently than predicted by the NCM are shown in different colors. $R^2$ indicates the fit to the model. **d**, The MENs of five water ecosystems in Tibet were constructed based on Spearman correlations of genera relative abundances produced by MetaPhlAn (version 4.0.1). Modules with ≥2 nodes are shown in different colors, and smaller modules are shown in grey. Details of network topological attributes are listed in **Supplementary Table 2**. **e**, The vulnerability variation of the MENs with a range of percent of nodes stripped. NCM, neutral community model; MEN, molecular ecological network.

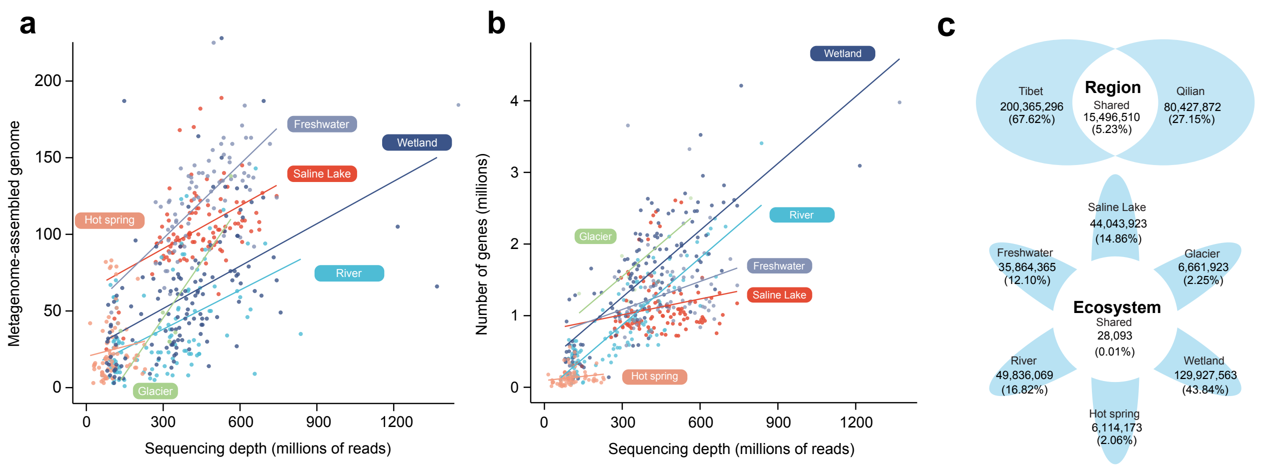


**Fig. S3 | The number of metagenome-assembled genomes and unigenes increase with increasing sequencing depth**. **a**, The correlation between the number of MAGs and the sequencing depth. At the same sequencing depth, freshwater lake and saline lake recover the greatest number of MAGs. **b**, The correlation between the number of unigenes and the sequencing depth. The color of the dots and lines are based on the water ecosystem type of the samples. **c**, The number of unigenes that are unique to each water ecosystem or region, as well as the number of unigenes that are shared by multiple water ecosystems or regions. MAG, metagenome-assembled genome.

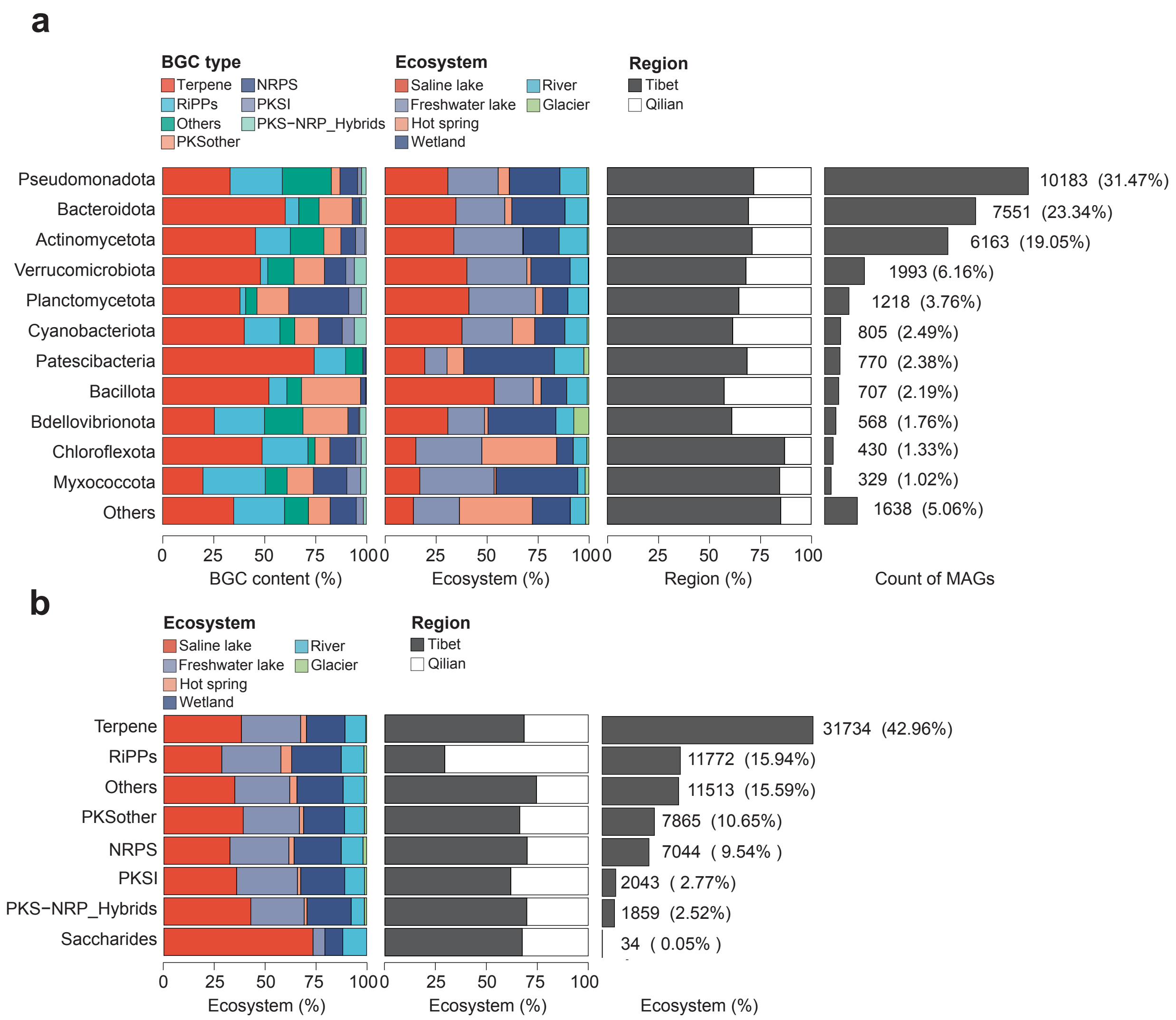


**Fig. S4 | Environmental and geographical distribution of metagenome-assembled genomes and biosynthetic gene clusters. a**, The relative frequency of BGC types across the phyla. The environmental and geographical distribution, and the proportion of the MAGs at the phylum level. Phyla with <1% number of MAGs were designated as “Others”. **b**, Environmental and geographical distribution, and the proportion of the BGC types. BGC, biosynthetic gene cluster; MAG, metagenome-assembled genome.

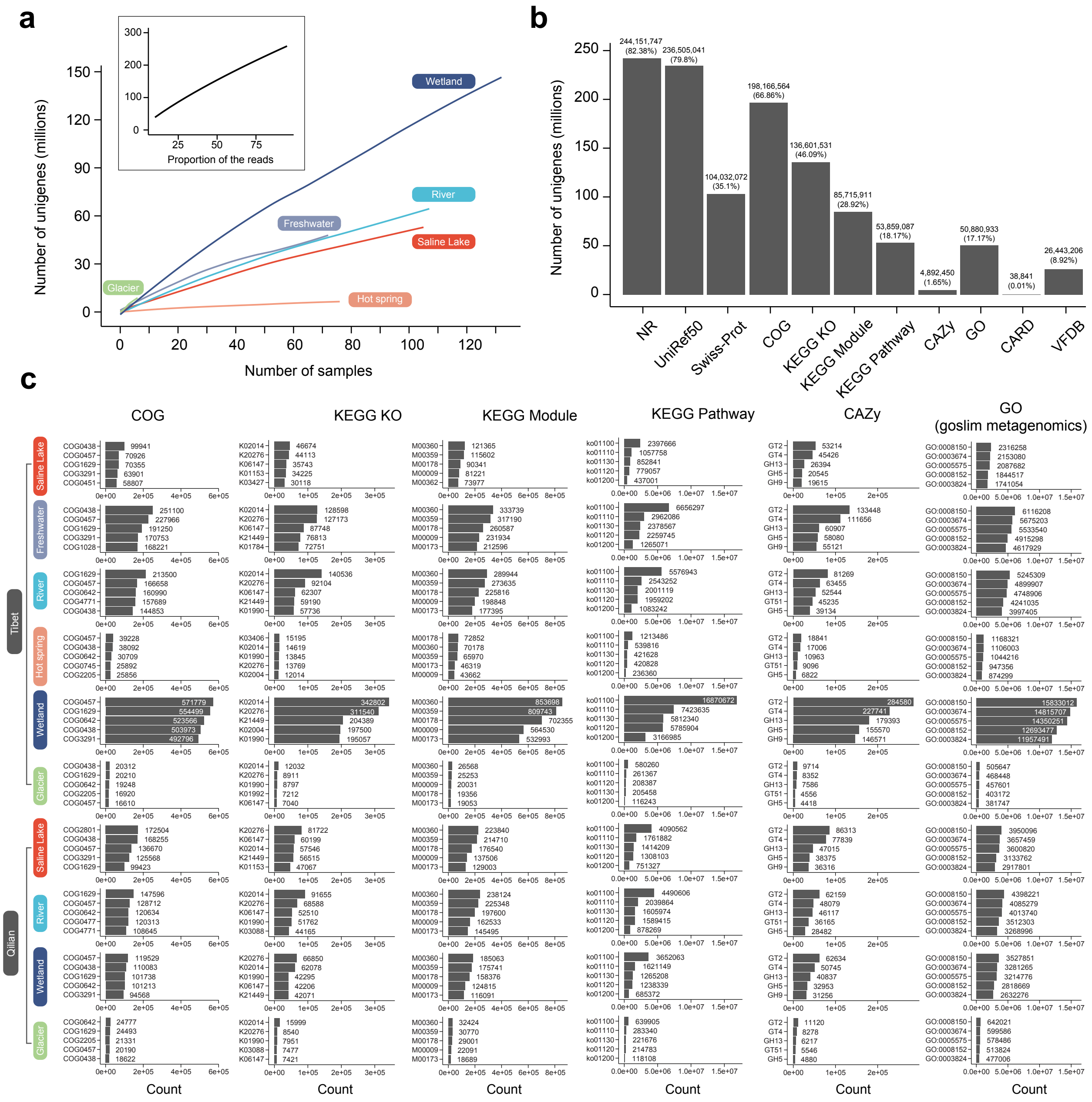


**Fig. S5 | Functional annotations of the TPMC gene catalog. a**, The unigene accumulation curves of each water ecosystem. **b**, The number of unigenes mapped to the databases. **c**, The top five enriched functions in each water ecosystem of Tibet and Qilian (**Supplementary Table 6**).

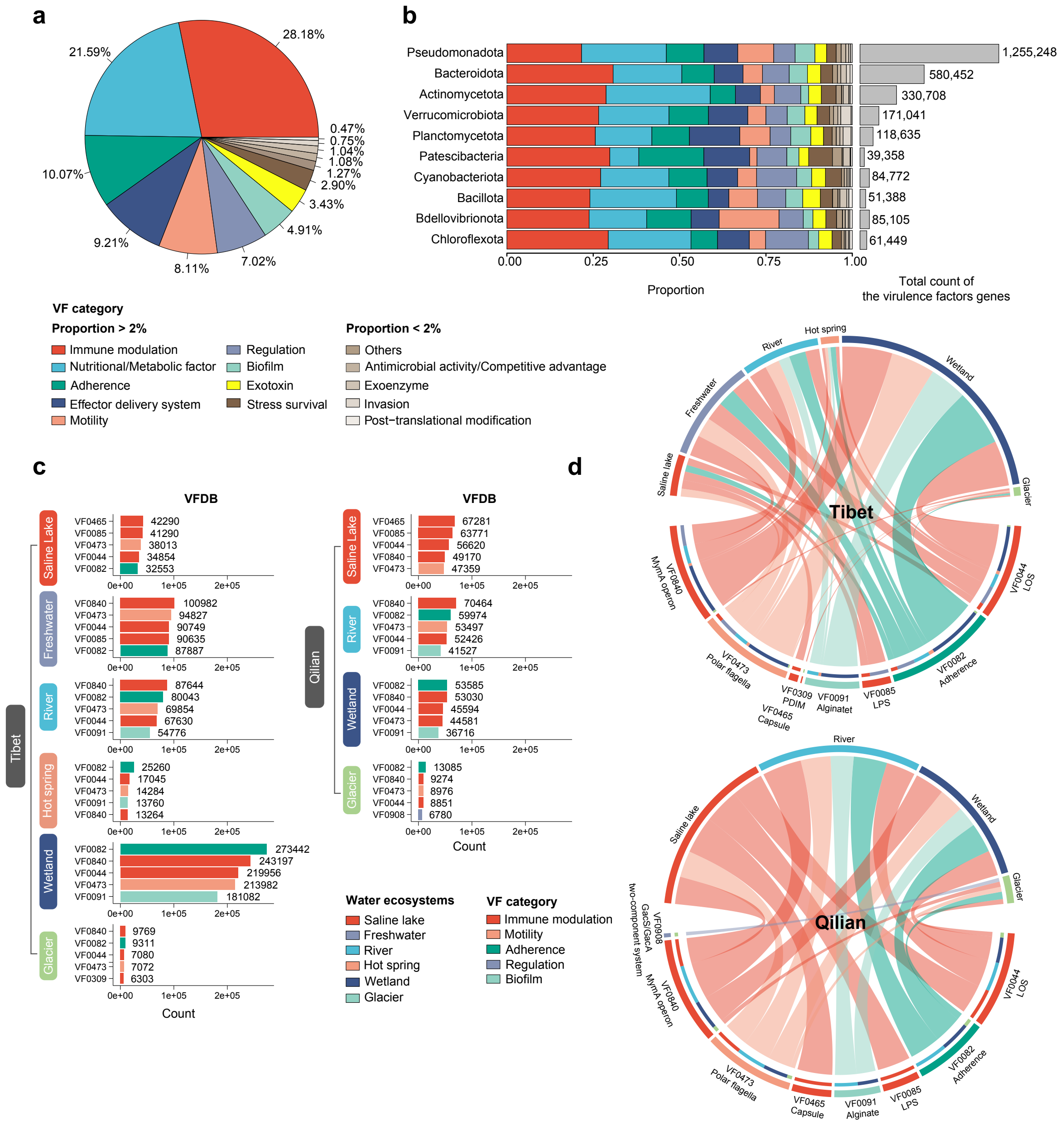

**Fig. S6 | TPMC harbor a diverse range of potential virulence factors across regions and water ecosystems. a**, The diversity and proportion of the VFs identified based on VFDB. **b**, The proportion of VF categories in the top ten MAGs (left) and the total count of the VFs in the MAG. **c**, The count of the top five enriched VFs in each water ecosystem of Tibet and Qilian (**Supplementary Table 6**). **d**, The contribution of each water ecosystem to the count of the VFs in the Tibet and Qilian. VF, virulence factor; MAG, metagenome-assembled genome.

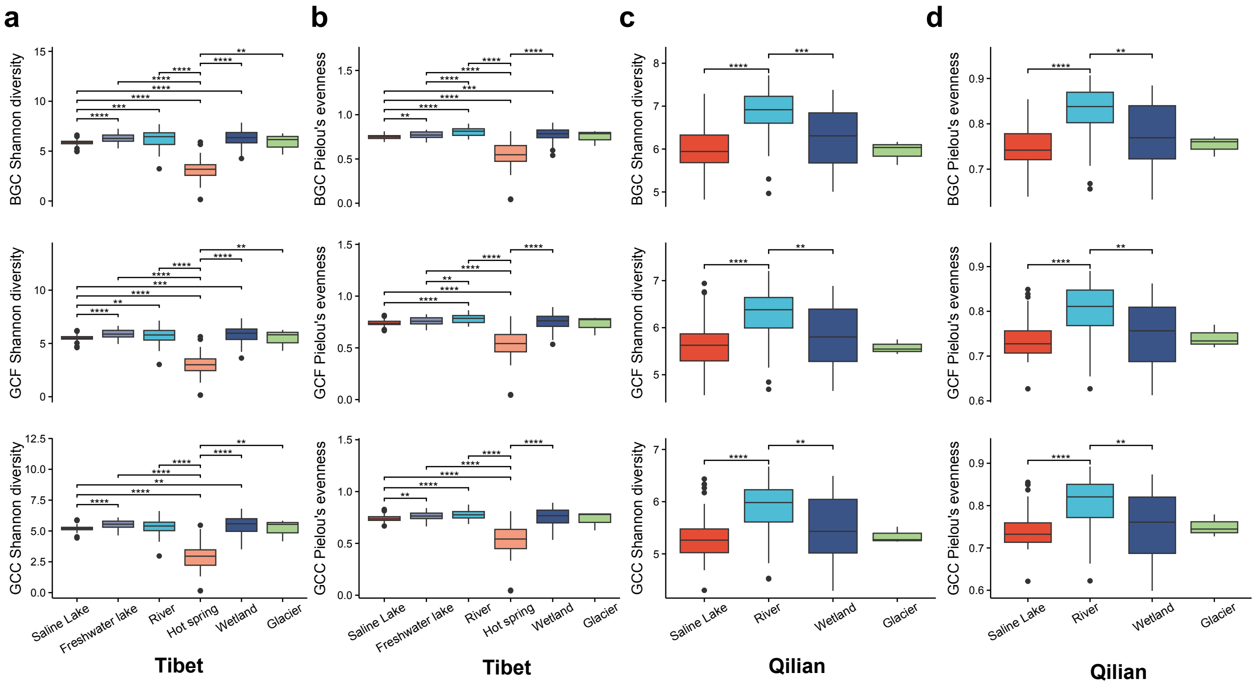


**Fig. S7 | Metagenomic profiles of the biosynthetic gene clusters.** The Shannon diversity and Pielou's evenness diversity of BGCs, GCFs, and GCCs across the water ecosystems. The statistical significance of differences in diversity between ecosystems is calculated by the two-sided Mann-Whitney-Wilcoxon test (*P < 0.05， **P < 0.01, ***P < 0.001, ****P < 0.0001). Boxes represent the interquartile range between the first and third quartiles and the line inside represents the median. Whiskers denote the lowest and highest values within the 1.5×interquartile range from the first and third quartiles, respectively. BGC, biosynthetic gene cluster; GCF, gene cluster family; GCC, gene cluster clan.

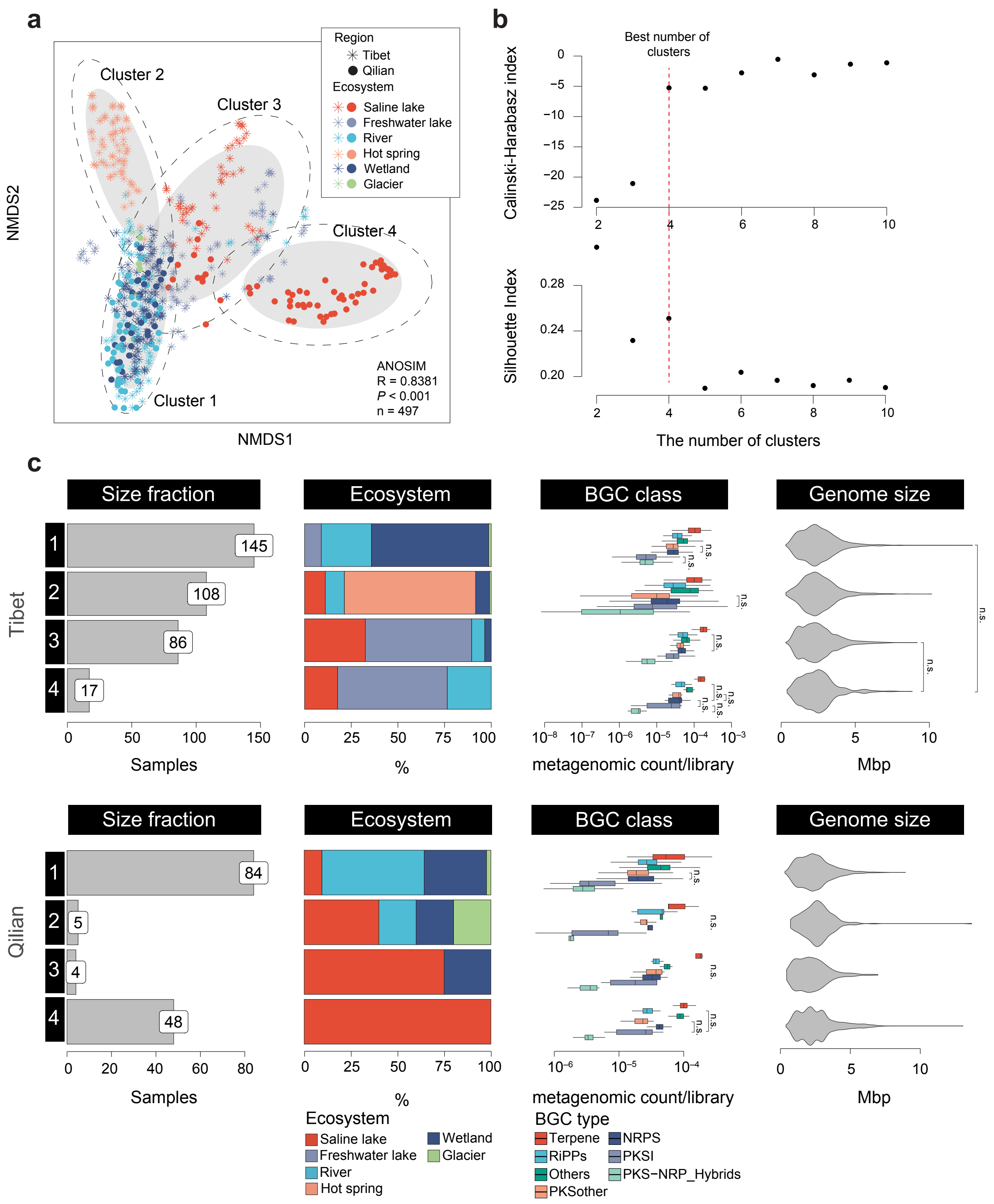


**Fig. S8 | Sample clustering based on metagenomic profiles of the biosynthetic gene clusters.**

**a**, The metagenomic abundances of GCCs (methods) are used to compute Jensen–Shannon

divergence distances among the 497 samples (One sample in TPMC has no predicted BGC). Using PAM and NMDS, we identify four sample clusters. ANOSIM tests are used to test the statistical significance of whether distances between clusters are greater than within clusters (R and *P*). **b**, Calinski-Harabasz index and Silhouette Index are used to evaluate the optimal number of clusters. **c**, These clusters are categorized by location, including size fractions and water ecosystems. Significant differences in BGC types and average genome sizes are calculated between the clusters. The statistical significance of the differences is calculated by the two-sided Mann-Whitney-Wilcoxon test. Pairs of boxplots and violin plots marked without n.s. are significant ($P < 0.05$). Boxes represent the interquartile range between the first and third quartiles and the line inside represents the median. Whiskers denote the lowest and highest values within the 1.5×interquartile range from the first and third quartiles, respectively. GCC, gene cluster clan; PAM, partition around medoids; NMDS, non-metric multidimensional scaling. ANOSIM, Analysis of similarities.

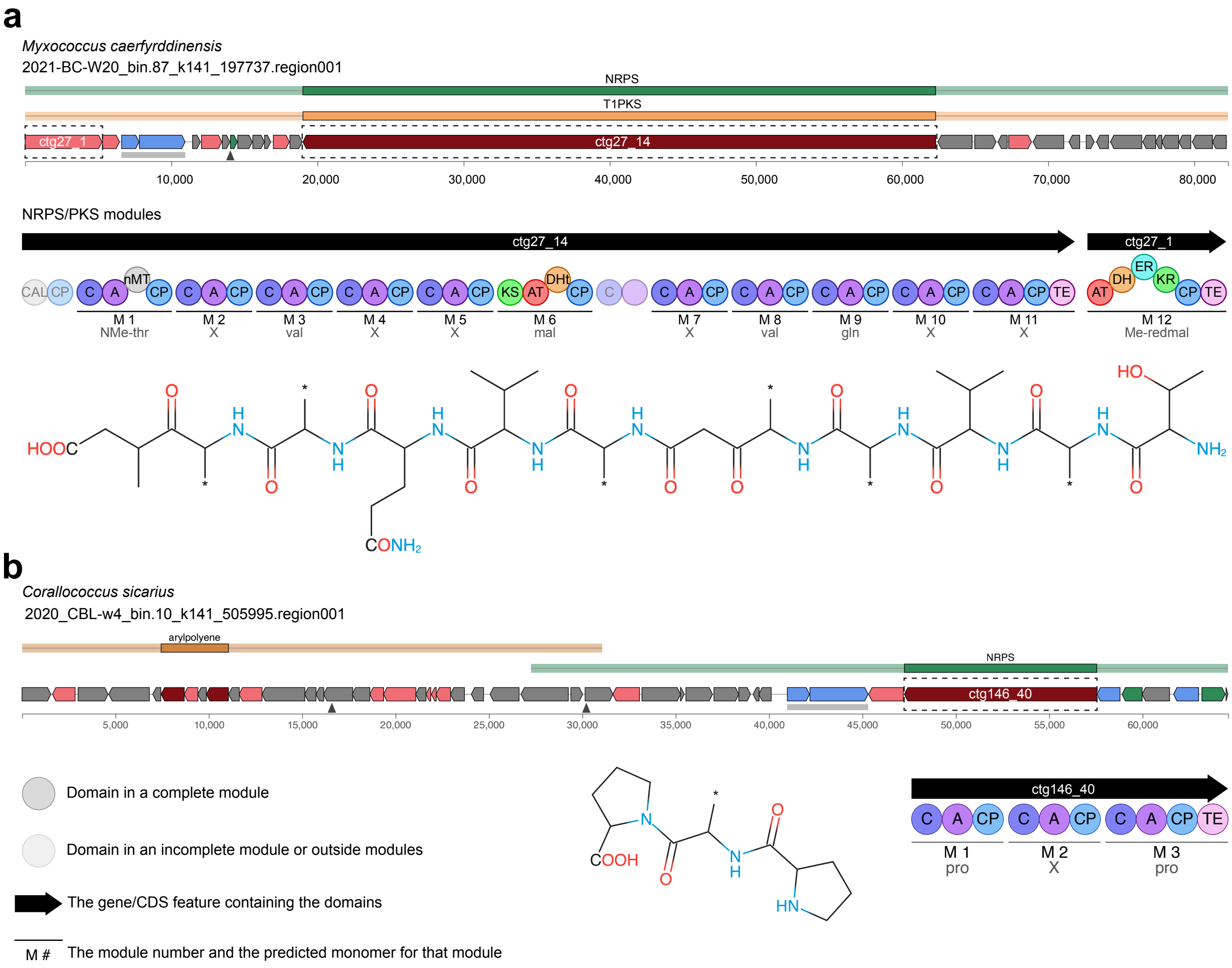


**Fig. S9 | Two biosynthetic gene clusters predicted from *Myxococcus caerfyrddinensis* and *Corallococcus sicarius*. a,** The longest BGCs (82,306) bp predicted from the MAG of species *Myxococcus caerfyrddinensis* that encodes NRPS and T1PKS, with 12 core modules and the predicted product structure displayed. **b**, The longest BGCs (64,575 bp) predicted from the MAG of species *Corallococcus sicarius* that encodes NRPS, with 3 core modules and the predicted product structure displayed. BGC, biosynthetic gene cluster; MAG, metagenome-assembled genome; NRPS, nonribosomal peptides; T1PKS, type I polyketide synthase.

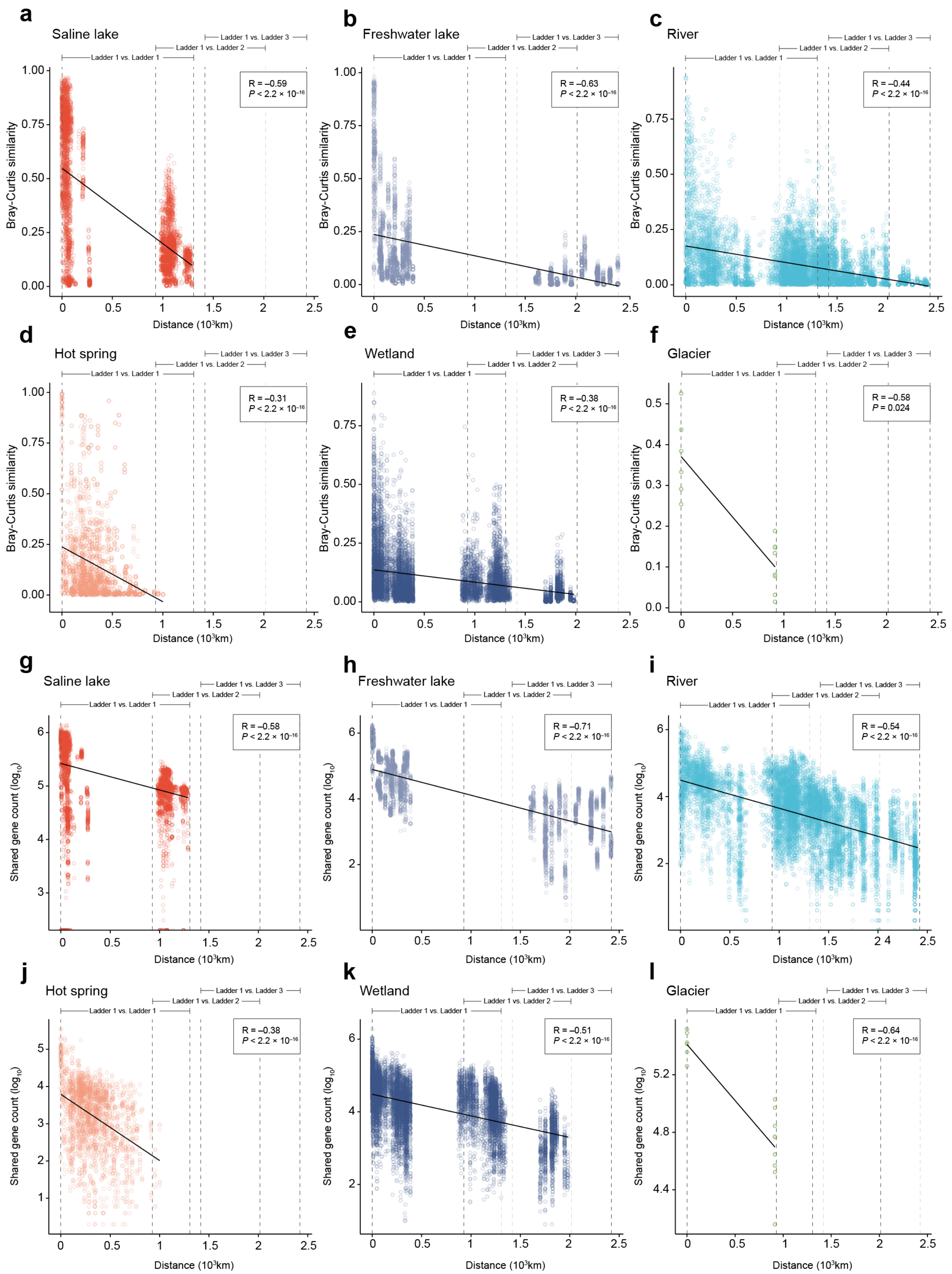

a
Saline lake
R = –0.59
P < 2.2 × 10⁻¹⁶
b
Freshwater lake
R = –0.63
P < 2.2 × 10⁻¹⁶
c
River
R = –0.44
P < 2.2 × 10⁻¹⁶
d
Hot spring
R = –0.31
P < 2.2 × 10⁻¹⁶
e
Wetland
R = –0.38
P < 2.2 × 10⁻¹⁶
f
Glacier
R = –0.58
P = 0.024
g
Saline lake
R = –0.58
P < 2.2 × 10⁻¹⁶
h
Freshwater lake
R = –0.71
P < 2.2 × 10⁻¹⁶
i
River
R = –0.54
P < 2.2 × 10⁻¹⁶
j
Hot spring
R = –0.38
P < 2.2 × 10⁻¹⁶
k
Wetland
R = –0.51
P < 2.2 × 10⁻¹⁶
l
Glacier
R = –0.64
P < 2.2 × 10⁻¹⁶
Ladder 1 vs. Ladder 1
Ladder 1 vs. Ladder 2
Ladder 1 vs. Ladder 3
Bray-Curtis similarity
Shared gene count (log10)
Distance (10³km)

**Fig. S10 | The microbial taxonomic and functional differences against geographical distances across a 2,500-km transect. a–f**, The microbial taxonomic profiles become less similar between samples in the same water ecosystem, as the distance increases. **g–l**, The number of the shared genes decreases between samples in the same water ecosystem, as the distance increases. Linear regression is performed to calculate the R and *P* values. For all the panels, the comparisons are conducted within the Tibetan Plateau, as well as between the Tibetan Plateau (Ladder 1) and the others (Ladder 2 and Ladder 3).

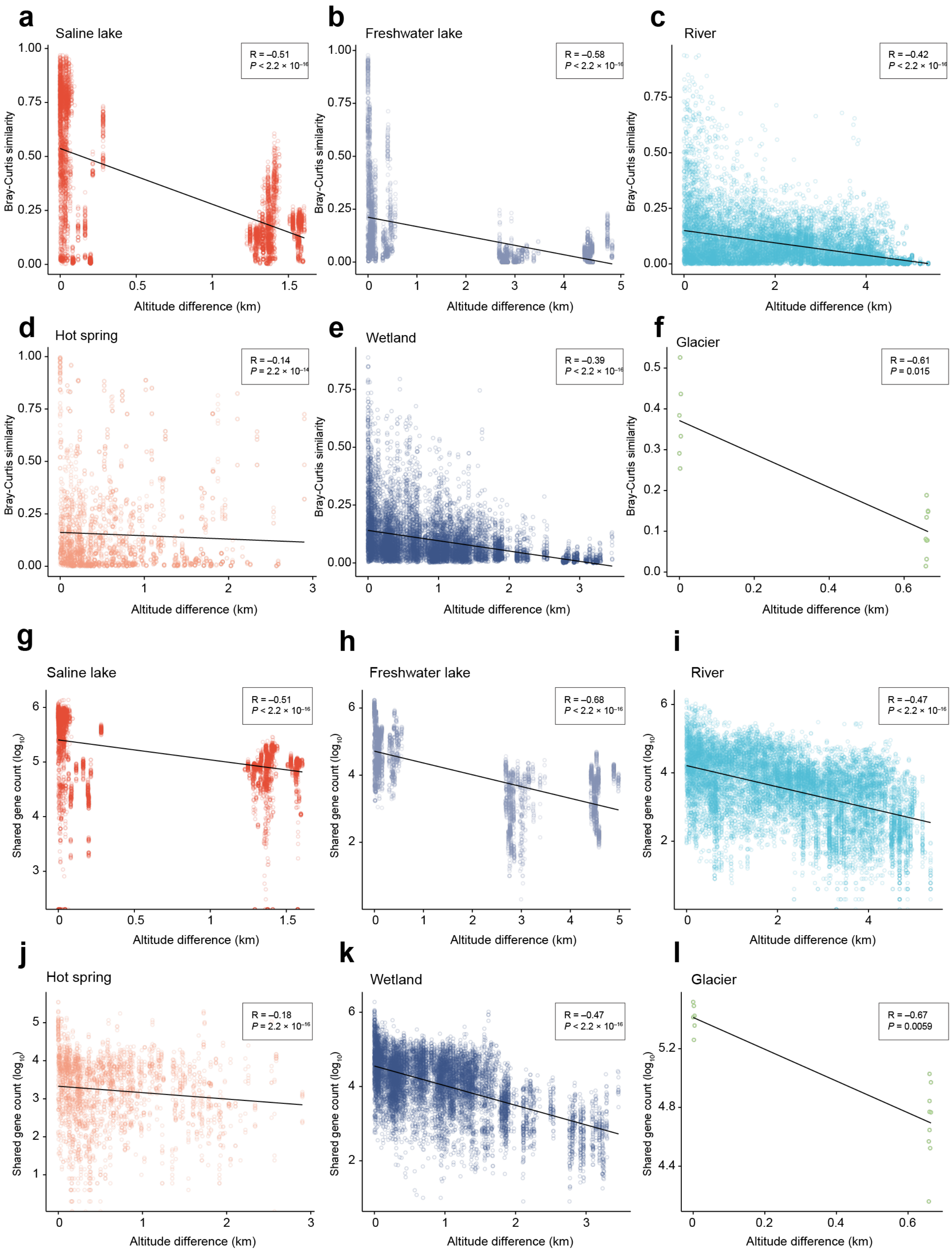
a
Saline lake
R = –0.51
P < 2.2 × 10–16
Bray-Curtis similarity
Altitude difference (km)
b
Freshwater lake
R = –0.58
P < 2.2 × 10–16
Bray-Curtis similarity
Altitude difference (km)
c
River
R = –0.42
P < 2.2 × 10–16
Bray-Curtis similarity
Altitude difference (km)
d
Hot spring
R = –0.14
P = 2.2 × 10–14
Bray-Curtis similarity
Altitude difference (km)
e
Wetland
R = –0.39
P < 2.2 × 10–16
Bray-Curtis similarity
Altitude difference (km)
f
Glacier
R = –0.61
P = 0.015
Bray-Curtis similarity
Altitude difference (km)
g
Saline lake
R = –0.51
P < 2.2 × 10–16
Shared gene count (log10)
Altitude difference (km)
h
Freshwater lake
R = –0.68
P < 2.2 × 10–16
Shared gene count (log10)
Altitude difference (km)
i
River
R = –0.47
P < 2.2 × 10–16
Shared gene count (log10)
Altitude difference (km)
j
Hot spring
R = –0.18
P = 2.2 × 10–16
Shared gene count (log10)
Altitude difference (km)
k
Wetland
R = –0.47
P < 2.2 × 10–16
Shared gene count (log10)
Altitude difference (km)
l
Glacier
R = –0.67
P = 0.0059
Shared gene count (log10)
Altitude difference (km)

**Fig. S11 | The microbial taxonomic and functional differences against altitude differences across a 2,500-km transect. a–f**, The microbial taxonomic profiles become less similar between samples in the same water ecosystem, as the altitude difference increases. **g–l**, The number of the shared genes decreases between samples in the same water ecosystem, as the altitude difference increases. Linear regression is performed to calculate the R and *P* values. For all the panels, the comparisons are conducted within the Tibetan Plateau, as well as between the Tibetan Plateau (Ladder 1) and the others (Ladder 2 and Ladder 3).

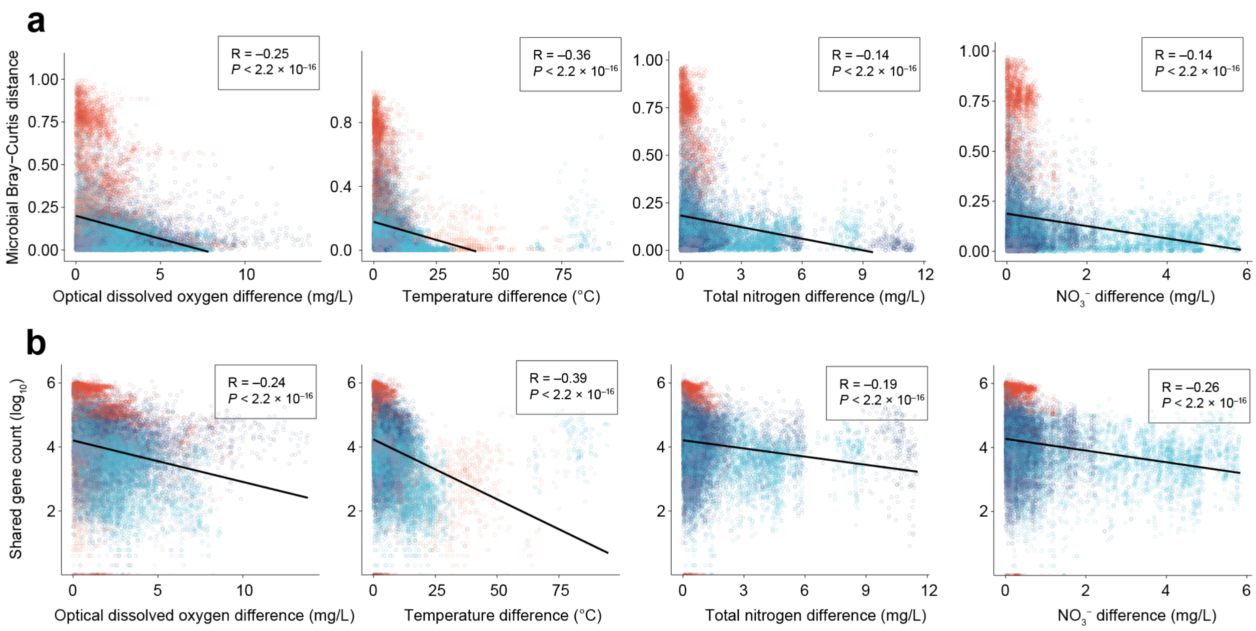


**Fig. S12 | Microbial taxonomic and functional differences against environmental differences across Chinese a 2,500-km transect. a–b**, As the environmental differences increase, the microbial taxonomic profiles become less similar (a), and the number of the shared genes decreases (b), between samples in the same water ecosystem. Linear regression is performed to calculate the R and *P* values. For all the panels, the comparisons are conducted within the Tibetan Plateau, as well as between the Tibetan Plateau (Ladder 1) and the others (Ladder 2 and Ladder 3).
