# Supplementary material for: A genome and gene catalog of the aquatic microbiomes of the Tibetan Plateau
Source: Nat Commun. 2024 Feb 16;15:1438. doi: 10.1038/s41467-024-45895-8 (PMC10873407; doi:10.1038/s41467-024-45895-8)
Supplement: Supplementary file 3 — Description of Additional Supplementary Files [file 41467_2024_45895_MOESM3_ESM.pdf]

File Name: Supplementary Data 1
Description: Characteristics of the metagenome-assembled genomes in the TPMC

File Name: Supplementary Data 2
Description: Supplementary tables of the TPMC, including 12 tabs as below:

Supplementary Data 2.1 Correlations between microbial diversity and environmental factors.

Supplementary Data 2.2 Network parameters of each water ecosystems.

Supplementary Data 2.3 Summary of the quality of the metagenome-assembled genomes grouped by ecosystems and regions.

Supplementary Data 2.4 The comparison between the representative TPMC MAGs to the representative genomes from GTDB, GEM, TG2G, and TARA catalogs.

Supplementary Data 2.5 Functional annotation of the gene catalog across regions and biomes. The top 20 annotations of each database are displayed.

Supplementary Data 2.6 Characteristics of the biosynthetic gene cluster.

Supplementary Data 2.7 The number of biosynthetic gene cluster in each phylum.

Supplementary Data 2.8 The geographical distribution of the number of biosynthetic gene clusters.

Supplementary Data 2.9 The novelty of the biosynthetic gene cluster.

Supplementary Data 2.10 The novelty of the gene cluster family of the biosynthetic gene cluster.

Supplementary Data 2.11 The novelty of the gene cluster clan of the biosynthetic gene cluster

Supplementary Data 2.12 The genome-based species presenting the highest biosynthetic potential.

File Name: Supplementary Information
Description: Supplementary Figs. 1–9
